# COVID-19: Test, Test and Test

**DOI:** 10.3390/medsci9010001

**Published:** 2020-12-30

**Authors:** Fatima A Saleh, Aleen Sleem

**Affiliations:** 1 Department of Medical Laboratory Technology, Faculty of Health Sciences, Beirut Arab University, Beirut 115020, Lebanon; 2 Department of Molecular Diagnostics, Doctors’ Center Laboratories, Beirut 115020, Lebanon; aleensleem29@gmail.com

**Keywords:** COVID-19, RT-PCR, diagnostic testing, sampling methods, nasopharyngeal swabs, false-negative results

## Abstract

A new virus was identified in late December 2019 when China reported the first cases of pneumonia in Wuhan, and a global COVID-19 pandemic followed. The world was not late to respond, with a number of sweeping measures ranging from social distancing protocols, stringent hygienic practices, and nation-wide lockdowns, as well as COVID-19 testing campaigns in an attempt to prevent the transmission of the disease and contain the pandemic. Currently, different types of diagnostic testing have been adopted globally, such as nucleic acid detection tests, immunological tests and imaging approaches; however, real-time reverse transcriptase–polymerase chain reaction (RT-PCR) remains the “gold standard” for detection of severe acute respiratory syndrome coronavirus 2 (SARS-CoV-2). Pre-analytical factors, such as specimen selection and collection, are crucial for RT-PCR, and any suboptimal collection may contribute to false-negative results. Herein, we address some of the specimen types that have been used in molecular detection methods for COVID-19. However, the pandemic is still evolving, and information might change as more studies are conducted.

## 1. Introduction 

**Figure 1:**
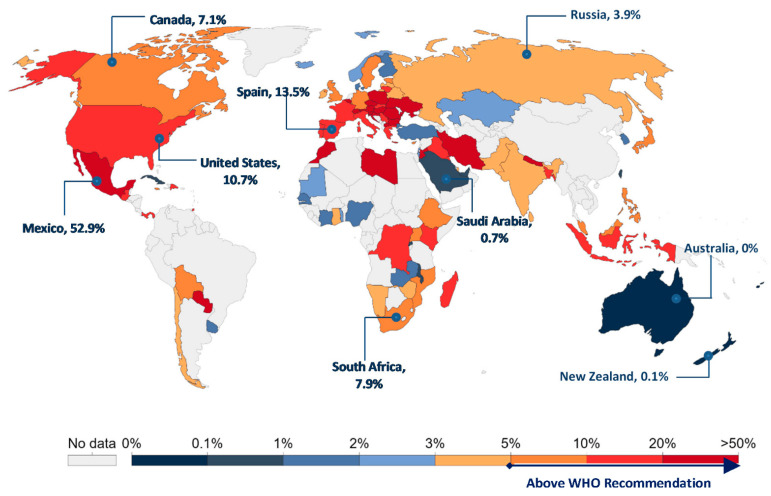
Worldwide map of seven-day average COVID-19 positivity rates as of 12 November 2020. Positivity rates are lowest in Australia and New Zealand and highest in countries like Mexico. Adapted from Our World in Data [6].

To date, severe acute respiratory syndrome coronavirus 2 (SARS-CoV-2) remains a global threat that is yet to be contained. As of 12 November 2020, there have been more than 51 million confirmed cases of COVID-19, with the pandemic claiming the lives of over 1.27 million people worldwide as reported by the WHO [1]. In the absence of vaccine and effective treatments, countries around the world have adopted different strategies to prevent the transmission of the virus and combat the pandemic [2]. These sweeping measures range from mandatory use of masks in public, stringent social distancing protocols, and partial or nation-wide lockdowns, as well as large-scale testing campaigns. Crucially, testing more people is essential to identify who is infected and, therefore, isolate and track them in order to avoid spreading the disease, thus temporarily filtering people with COVID-19 out of the population, including asymptomatic individuals. The WHO director even called upon all countries to “test, test, test” in order to battle the pandemic that swept the globe [3]. The testing, tracking, and tracing (TTT) approach was therefore adopted, implemented and scaled-up by many countries. The TTT strategy is not a new one, as it has been used before and proved its effectiveness in other outbreaks, such as the 2014 Ebola virus outbreak, the outbreak of Middle East respiratory syndrome (MERS) in 2012, and in the severe acute respiratory syndrome (SARS) outbreak in 2003 [4]. As for COVID-19, while many countries are struggling to enforce public health recommendations despite individuals’ desire for autonomy and in-person communication, countries such as Singapore, South Korea and New Zealand have used the TTT approach as part of their strategy to control the pandemic for a longer period of time [5]. Herein, as shown in Figure 1, these countries have scored a positivity rate of less than 1%. As published by the WHO, a positivity rate of less than 5% is an indication that the pandemic is well under control [1].

## 2. COVID-19 Diagnostic Testing

Currently, there are several types of diagnostic tests that have been endorsed by the WHO, and healthcare systems around the world, such as nucleic acid detection tests, immunological tests and imaging approaches; however, to date real-time reverse transcriptase–polymerase chain reaction (RT-PCR) remains the ”gold standard” for the detection of SARS-CoV-2, which bears a single stranded RNA genome [7]. Molecular laboratory departments have been overloaded with specimens for RT-PCR tests amid the current pandemic. RT-PCR is a nucleic assay that works by copying a very small number of viral RNA strands into billions that can be easily detected. The envelope protein (E), nucleocapsid protein (N) and RdRp (RNA-dependent RNA polymerase) genes are the main three regions of the SARS-CoV-2 genome targeted by RT-PCR. Among these, detecting the RdRp gene has the highest analytical sensitivity [8]. Many studies have also stressed the importance of dual target testing, using the E gene as a second target, to help reduce the risk of low sensitivity due to mutation and/or low viral loads and to improve the clinical response to the COVID-19 pandemic [9,10]. The European Centre for Disease Prevention and Control (ECDC) has advised against E gene RT-PCR in isolation because of non-specificity concerns and contamination issues [11].

A serious concern for the RT-PCR testing is the risk of false-negative and false-positive results, and there are many studies in the literature documenting false-negative RT-PCR tests [12,13]. Li et al. reported a high false-negative rate of RT-PCR results when studying a total of 610 hospitalized COVID-19 patients from Wuhan, China [13]. Sample selection and collection among other pre-analytical factors are crucial, and any suboptimal collection of specimen may contribute to false-negative results.

## 3. Sampling Methods

Upper respiratory tract samples such as nasopharyngeal swabs have been recommended by several studies that examined the viral load of SARS-CoV-2 patients and are the most commonly used collection method worldwide. A recent publication by Zou et al. compared the aggregated cycle threshold (Ct) of nasal (mid-turbinate and nasopharyngeal) and throat (oropharyngeal) swabs of 14 patients [14]. Findings revealed that the Ct value of PCR was 21 with nasal swabs, which is significantly lower than the Ct value of 40 with throat swabs for COVID-19 patients 2 days after the onset of symptoms, suggesting that an accurate viral load could be obtained from a nasal swab rather than a throat swab [14]. Another study compared the performance between nasopharyngeal and oropharyngeal swabs for SARS-CoV-2 detection in 353 COVID-19 patients [15]. Results showed a higher positivity rate in nasopharyngeal samples than oropharyngeal ones, suggesting the former as the preferred collection method over the latter [15]. Correct collection of samples is critical, and healthcare workers must be well trained prior to collection to ensure accurate results. On a technical note, nasopharyngeal swabs must be inserted horizontally, parallel to the palate, until no further insertion is possible; the swab must be twirled and left for a couple of seconds to absorb the fluids [16]. To minimize incorrect sample collection and to overcome the global shortage in nasopharyngeal swabs, scientists tested the feasibility of using nasal sampling as a less invasive alternative source [17,18]. A study by Péré and colleagues demonstrated that SARS-CoV-2 molecular testing from nasal swabs showed high sensitivity (89.2%) and specificity (100%) that were nearly equivalent to using nasopharyngeal swabs, suggesting the possibility of relying on them for substitution [18].

Additionally, saliva is emerging as a promising alternate source for SARS-CoV-2 diagnosis. Indeed, the use of saliva offers many advantages over nasopharyngeal or oropharyngeal swabs, such as non-invasiveness, cost effectiveness and possibility of self-collection, thus minimizing the exposure of healthcare workers to infected patients [19,20,21]. Moreover, studies have reported saliva specimens to have almost comparable sensitivity 91% (95% CI: 80–99%) and specificity 97.6% (95% CI: 95.5–98.9%) to “gold standard” nasopharyngeal sampling in the detection of SARS-CoV-2 (Table 1) [19,20,21].

Due to the worldwide demand for extraction kits and reagents, pooling of nasopharyngeal swabs has been used to compensate for the shortage [22]. Pooling is a grouped extraction for 3–5 samples in one tube following similar extraction procedure. Amplification indicates the presence of one or more positive samples within the group; individual extraction of the group identifies the positive samples. Absence of amplification indicates that all of the grouped samples are negative, making pooling a cost effective and applicable procedure [23]. However, inaccurate results have been identified in more than one area within the process, particularly the high risk of false-negative results for borderline positive patients with a high Ct value (low viral load) due to viral dilution [24]. Nevertheless, this technical limitation could be overcome by using highly sensitive kits with additional thermal cycles to reduce the possibility of missing “weak positive” COVID-19 test results [24,25].

Lower respiratory tract specimens, such as sputum, although not always available, are optimal for diagnosing severely ill COVID-19 patients, providing a high sensitivity of 97.2% (95% CI: 90.3–99.7%) [26]. In a study that investigated how long the virus remains in the body of COVID-19 patients post-recovery after two consecutive negative pharyngeal tests, 22 out of 133 patients showed positive results for the presence of SARS-CoV-2 in sputum after 39 days of testing negative with a pharyngeal swab [27]. Moreover, Pan et al. reported higher viral loads in sputum samples than in throat swab samples [28]. These findings raise doubts about the results that are being reported in laboratories daily and bring into question whether negative patients are truly low risk to the people around them or not [27].

During the early days of the pandemic, the swabs were initially placed in viral transport medium (VTM); however, its shortage has made it essential to develop alternatives to be used immediately. Studies have compared the viability of the SARS-CoV-2 in different transporting media such as phosphate-buffered saline (PBS), 0.9% saline, and minimum essential medium (MEM). Forty-eight positive samples were placed in VTM, MEM, PBS, and 0.9% saline. All samples spiked amplification and tested positive in all media. Half of the samples were later frozen and the other half was stored at 2–8 °C and then tested at day 1, 3, and 7; all showed positive results [29]. This study provides sensitive and approved alternatives for VTM, thus solving the shortage for optimal sample collection and transportation [29].

Other studies have investigated the presence of SARS-CoV-2 RNA in the feces of COVID-19 patients. Although viral load in the fecal specimens was shown to be lower than the upper respiratory tract specimens during initial onset of symptoms; fecal specimens have shown promising results in detecting the virus [28]. Previously infected patients that have tested negative nasally may show positive results from a fecal sample for several days after recovery [30]. Chen and colleagues showed that more than 60% of laboratory-confirmed COVID-19 patients tested positive for viral RNA in the feces even after the pharyngeal swabs turned negative twice [31]. However, the infectivity of viral RNA present in feces remains debatable. A study by Wölfel et al. failed to detect infectious virus from stool samples despite the high concentrations of virus RNA [32].

There have been reports that SARS-CoV-2 RNA might be detected in urine. A study conducted on 20 COVID-19 patients admitted to the National Center for Global Health and Medicine in Japan showed that the virus was only detected in the urine specimens of 2 patients (10%) [33]. In another study, urine samples collected from 72 COVID-19 patients from multiple sites in China tested negative for SARS-CoV-2 RNA [34]. However, the small number of patients enrolled and the lack of several clinical information are some of the limitations of these studies. Therefore, urine samples for SARS-CoV-19 diagnosis are out of consideration until further studies are conducted.

## 4. Conclusion

Taken as a whole, optimal sample collection is essential to avoid false-negative test results with consequences, including infected patients going unnoticed and therefore not isolating, subsequently leading to further spread of the pandemic.

## Figures and Tables

**Table 1:** Characteristics of different sampling methods for diagnosis of COVID-19.

Specimen	Advantages	Disadvantages	Sensitivity ^a^	Specificity	Ref.
Nasopharyngeal swabs (NPS)	Gold standard	Supervised sample collection, requires specialized medical personnel with PPE, expensive, reflex sneezing/coughing, high risk of viral transmission, patient discomfort	98% (CI: 89–100%)	98.1% (CI: 96.5–99.0%)	[15,35,36]
Oropharyngeal swabs (OPS)	High sensitivity if performed along with NPS	Supervised sample collection, requires specialized medical personnel with PPE, expensive, highest rate of aerosol transmission, more likely to have nausea and vomit, reflex sneezing/coughing, patient discomfort	21.1% (CI: 10.5–31.6%)	97.6% (CI: 93.9–99.5%)	[15,35,37,38]
Nasal swabs	Less invasive, less expensive, self-collection, no patient discomfort	Less accurate	87.1% (CI: 79.57–93.55%)	100% (CI: 69.2–100%)	[18,39,40,41]
Saliva	Self-collection, easy to obtain, cheap, non-invasive, low rate of aerosol transmission, cost-effective, does not require healthcare workers or PPE, no patient discomfort	Relatively less sensitive than NPS	91% (CI: 80–99%)	97.6% (CI: 95.5–98.9%)	[19,20,36,42,43]
Sputum	Less invasive than NPS, painless	Not all patients can provide it	97.2% (CI: 90.3–99.7%)	90.0% (CI: 73.5–97.9%)	[26,37,41,44]

^a^: sensitivity and specificity values are for real-time reverse transcriptase–polymerase chain reaction (RT-PCR) tests, and they are highly variable. Many factors can affect these values, such as time of sampling or clinical characteristics of patients (symptomatic or asymptomatic). CI: 95% Confidence Interval, PPE: Personal Protective Equipment.

## Data Availability

No new data were created or analyzed in this study. Data sharing is not applicable to this article.
